# The Evolution of Cell Communication: The Road not Taken

**DOI:** 10.4137/cci.s2776

**Published:** 2009-09-09

**Authors:** J.S. Torday, V.K. Rehan

**Affiliations:** Department of Pediatrics, Los Angeles Biomedical Research Institute at Harbor-UCLA Medical Center, Torrance, California, U.S

**Keywords:** cell communication, evolutionary biology, lung development, predictive medicine, preventive medicine, biologic space-time continuum

## Abstract

In the post-genomic era the complex problem of evolutionary biology can be tackled from the top-down, the bottom-up, or from the middle-out. Given the emergent and contingent nature of this process, we have chosen to take the latter approach, both as a mechanistic link to developmental biology and as a rational means of identifying signaling mechanisms based on their functional genomic significance. Using this approach, we have been able to configure a working model for lung evolution by reverse-engineering lung surfactant from the mammalian lung to the swim bladder of fish. Based on this archetypal cell-molecular model, we have reduced evolutionary biology to cell communication, starting with unicellular organisms communicating with the environment, followed by cell-cell communication to generate metazoa, culminating in the communication of genetic information between generations, i.e. reproduction. This model predicts the evolution of physiologic systems-including development, homeostasis, disease, regeneration/repair, and aging- as a logical consequence of biology reducing entropy. This approach provides a novel and robust way of formulating refutable, testable hypotheses to determine the ultimate origins and first principles of physiology, providing candidate genes for phenotypes hypothesized to have mediated evolutionary changes in structure and/or function. Ultimately, it will form the basis for predictive medicine and molecular bioethics, rather than merely showing associations between genes and pathology, which is an unequivocal Just So Story. In this new age of genomics, our reach must exceed our grasp.

“Two roads diverged in a wood, and I—

I took the one less traveled by,

And that has made all the difference.”

—Robert Frost, The Road Not Taken

**Preamble:** In his book Consilience,^1^ E.O. Wilson suggests that since all human knowledge is being reduced to computerized 1's and 0's, we can create a common database across all disciplines. In so doing, Wilson challenges us to generate a unifying theory for biology in order to fulfill this promise, because without such a theory, we only have disorganized information and anecdotes.^2^

What is the significance of Cell Communication? On its surface, it helps us to gain an understanding of complex biologic processes such as development, homeostasis, regeneration/repair and aging. But more importantly, cell communication is the essence of the evolutionary biologic process. Evolution can be reduced to communication- 1) between unicellular organisms and their physical environment, 2) the cell communications that form the basis for multicellularity, 3) communication of genetic material from one generation to the next, i.e. reproduction. This concept is distinguished from the writings of Jablonka and Lamb,^3^ or of Maynard Smith and Eors Szatmary,^4^ who focus on information, not on the evolved process of communication.

There have been many attempts to integrate biologic disciplines, beginning with the concept of the Great Chain of Being, Linnaeus's binomial nomenclature, and Darwin's Origin of Species.^5^ But more recently, it would seem that the closer we get to the basic elements of biology, the more skepticism we have experienced that we can achieve a synthesis. Witness the essay on “Life's Irreducible Structure” by Polanyi,^6^ or Prigogine's assessment of life's complexity,^7^ in which each of them independently concludes that biology is too complicated to define. In the midst of the sea change in biology we are now experiencing in the post-genomic era, it is helpful to step back and recalibrate in order to gain perspective on the processes of biology. The major take home message of the Human Genome Project was that humans have fewer genes than a carrot (25,000 vs. 40,000), whereas it had been predicted that we would have at least 100,000 genes, based on the number of genes found in worms, flies and the like- so much for a predictive paradigm. The fact that we humans have fewer genes doesn’t mean we are ‘simpler’ than organisms with more genes. It is more likely that we have used them more effectively as a result of evolutionary processes. Though we don't know what the mechanism of evolution is, our laboratory has gained some insight through a developmental cell-molecular approach to lung evolution.^8^

## Einstein's Vision of the Universe and the Darwinian Biologic Space-Time Continuum

**Figure 1:**
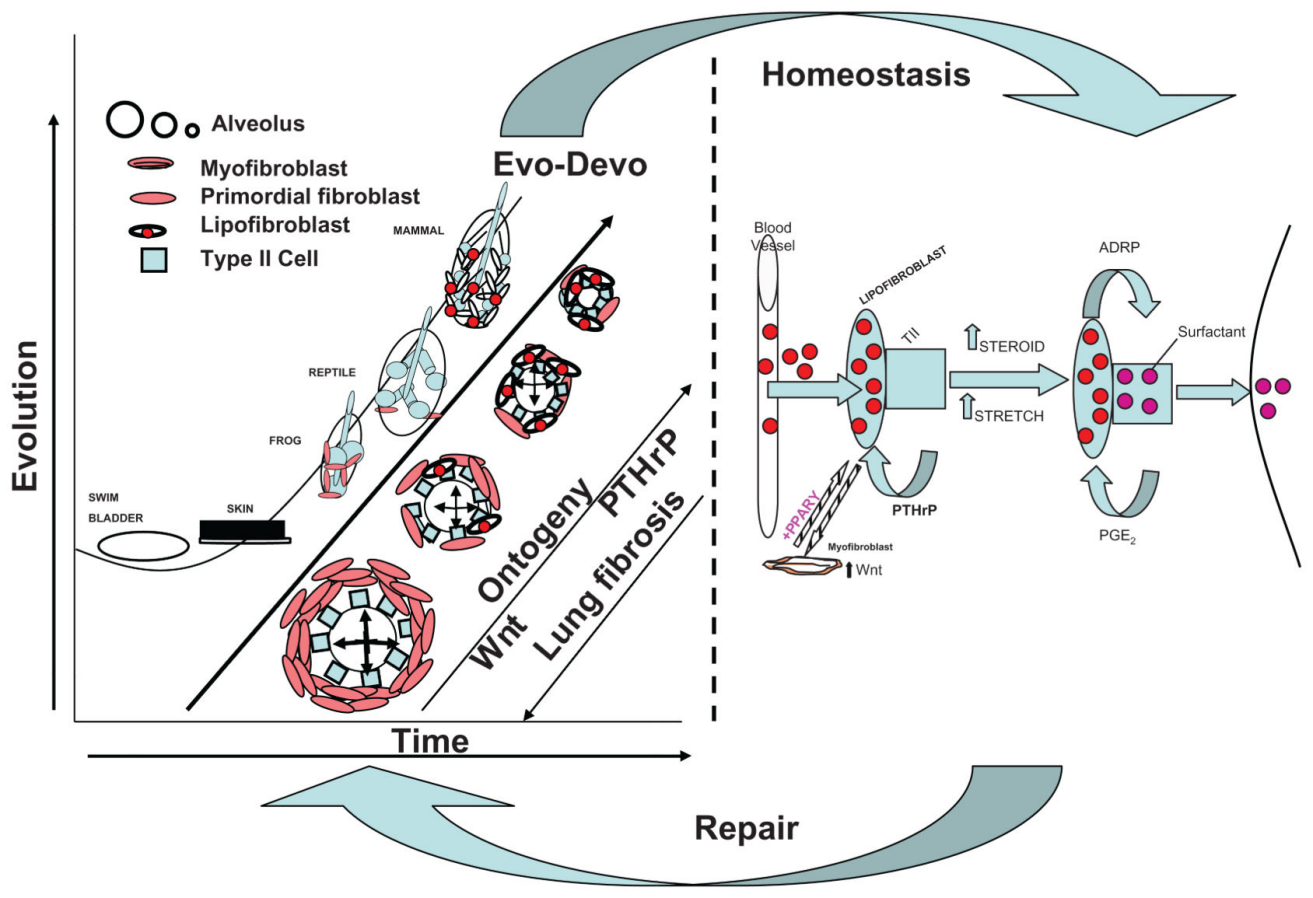
Lung biologic continuum from ontogeny-phylogeny to homeostasis and repair The schematic compares the cell-molecular progression of lung evolution from the fish swim bladder to the mammalian lung (left panel) with the development of the mammalian lung, or evo-devo, as the alveoli get progressively smaller (see Legend in upper left corner), increasing the surface area-to-blood volume ratio. This is facilitated by the decrease in alveolar myofibroblasts, and the increase in lipofibroblasts, due to the decrease in Wnt signaling, and increase in PTHrP signaling, respectively. Lung fibrosis progresses in the reverse direction (lower left corner). Lung homeostasis (right panel) is characterized by PTHrp/leptin signaling between the type II cell and lipofibroblast, coordinately regulating the stretch regulation of surfactant production and alveolar capillary perfusion. Failure of PTHrP signaling causes increased Wnt signaling, decreased PPARγ expression by lipofibroblasts, and transdifferentiation to myofibroblasts, causing lung fibrosis. Repair (arrow from homeostasis back to ontogeny/phylogeny), is the recapitulation of ontogeny/phylogeny, resulting in increased PPARγ expression.

Darwin saw a continuum of speciation based on principles of Natural Selection, not the anthropocentric Great Chain of Being. However, Darwin's explanation for the biologic patterns he observed was survival of the fittest, which is a metaphor for the evolutionary process, but does not provide a way of drilling down to the cell/molecular origins of life. Such a mechanistic model is necessary if we are going to take full advantage of the Human Genome and the genomes of other model organisms. For example, the cell-molecular mechanism of lung evolution depicted in the accompanying schematic (Fig. 1) infers that there is a ‘continuum’ from development to homeostasis and regeneration/repair. This depiction of the process of lung evolution, like a cladogram,^9^ also infers a direction and magnitude of change. That perspective is not unlike Einstein's vision as a 16 year old of traveling in parallel with a light beam through space, which gave him the insights to the physical continuum from Brownian Movement to the Photoelectric Effect and Relativity Theory.^10^ The space-time continuum that emerged from that epiphany has similarities to the accompanying schematic (Fig. 1) of the space-time continuum of lung biology, i.e. seen from a cell-cell signaling perspective, lung ontogeny, phylogeny, homeostasis and regeneration are a continuum. And since this model is largely based on universal developmental principles, the evolution of all other tissues and organs is amenable to the same mechanistic approach.

## Reverse Engineering of Physiologic Traits as a Portal for Viewing Evolution

The premise of the approach we have taken to evolution is that by tracing the ligand-receptor cell communications that determine the pulmonary surfactant ‘phenotype’ backward in time and space developmentally, both within and between species, we would be able to understand the mechanisms that have fashioned the lung through selection pressure.^11^ We have referred to this as a ‘middle-out’ approach, in contrast to the traditional top-down or bottom-up strategies. Horowitz^12^ formulated a similar approach to the evolution of biochemical pathways by assuming a retrograde mode of evolution, as follows: he envisioned an organism that could not synthesize an essential biochemical substance, so it acquired it from the environment. When the supply of that substance in the environment was exhausted, those organisms that possessed the last enzyme in the biosynthetic pathway could make use of the immediate precursor and convert it to the end product, until the supply of the immediate precursor was also exhausted. Then only those organisms that possessed the second to last enzyme could survive, and so on and so forth, until the biosynthetic pathway was completely established. This approach describes the functional phenotype for the evolution of a biosynthetic pathway, whereas the cell-molecular, middle-out approach would provide the series of ligand-receptor homeostatic mechanisms that determined those biosynthetic pathways from phenotypes to genes. Selection pressure on such ligand-receptor Gene Regulatory Networks generated both evolutionary stability and novelty through gene duplication, gene mutation, redundancy, alternative pathways, compensatory mechanisms, positive and balancing selection pressures, etc. Such genetic modifications were manifested by the structural and functional changes in the blood-gas barrier, primarily the thinning of the blood-gas barrier in combination with adaptive phylogenetic changes in the composition of the surfactant, as described eloquently by Daniels and Orgeig.^13^ The reverse engineering of these phenotypic changes in the blood-gas barrier form the basis for our molecular genetic approach to lung evolution.

## Cell Communication as the Basis for the Evolution of Metazoans

**Figure 2:**
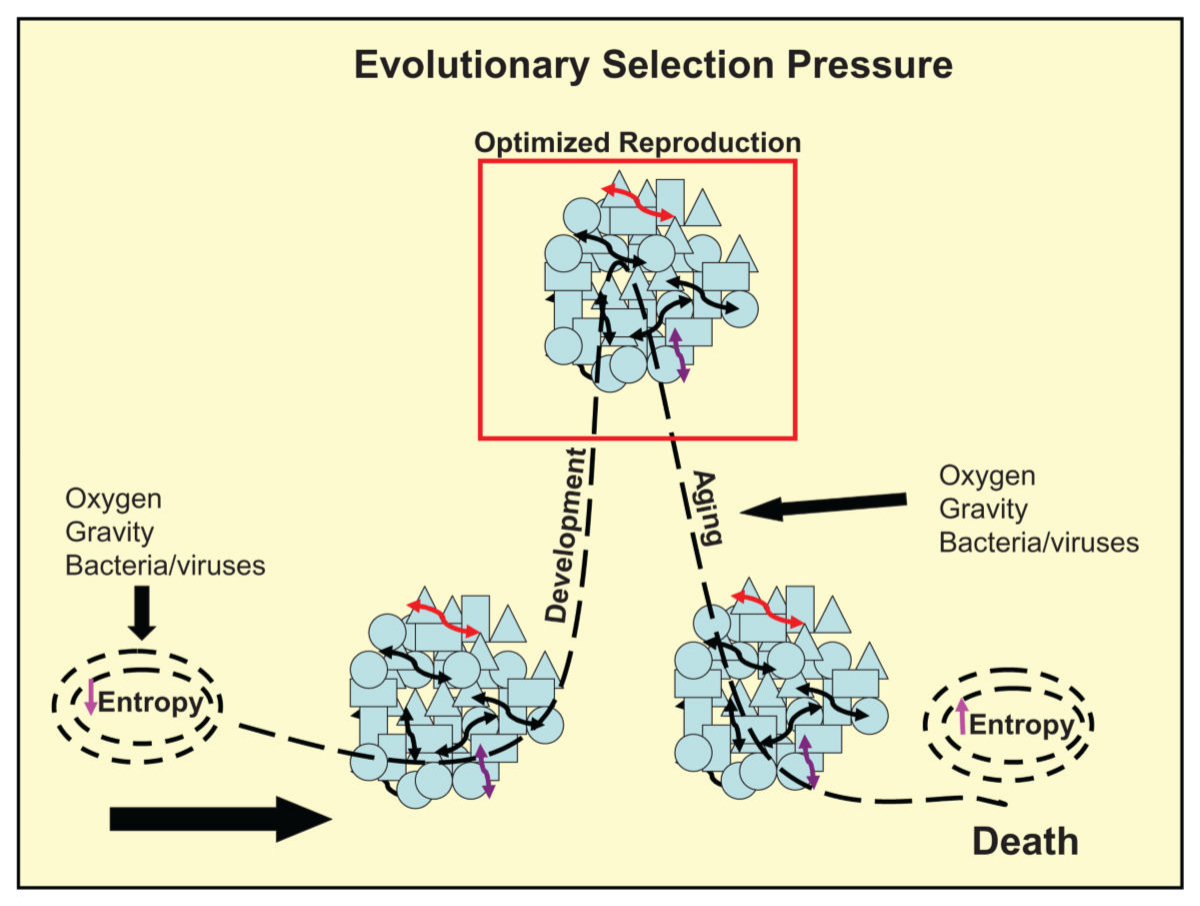
Evolutionary Selection Pressure, Development, Homeostasis, and Aging The schematic depicts the evolution of vertebrates, starting with the generation of micelles (far left), or semipermeable membrane spheres in which entropy decreased due to catalysis. selection pressure due to external forces (oxygen, gravity, bacteria, viruses) gave rise to multicellular organisms through cell communication (depicted by arrows between cells). Cell communication evolved into mechanisms of development, homeostasis and repair, optimized (red box) for reproduction. eventually this system fails as ‘pay back’ for defying the 2nd Law of Thermodynamics, resulting in decreased cell communication (Aging) and increased entropy (death).

Based on the middle-out cell communication model, how might biologic evolution have begun? One school of thought is that cellular organisms emerged as a result of the wetting and drying of lipids due to the diurnal rhythms of the sun, which gave rise to micelles, semi-permeable lipid membranes that would have provided a protected environment for the reduction of entropy through enzymatic catalysis (Fig. 2). Over the ensuing 4.5 billion years, such unicellular organisms have evolved in adaptation to their physical surroundings, eukaryotes evolving from prokaryotes, the former being distinguished from the latter by the presence of a nuclear envelope. That perinuclear membrane may well have evolved to protect the eukaryotic nucleus against invasion by prokaryotes.^14^ And the eukaryotic acquisition of mitochondria from prokaryotes, known as the Endosymbiosis Theory,^15^ similarly emerged as a result of the on-going competition between pro- and eukaryotes. Then, about 500 million years ago, unicellular organisms began cooperating with one another metabolically. In the case of the prokaryotes this took the form of such phenomena as biofilm and quorum sensing. Those ‘inventions’, in turn, may have been the selection pressure for eukaryotes to cooperate (or become extinct) by evolving the cell-cell signaling mechanisms that we recognize today as the soluble growth factors that mediate morphogenesis, homeostasis, regeneration and reproduction.^16^ However, it should be borne in mind that this process began with a decrease in entropy, defying the 2nd Law of Thermodynamics, fending off physical (gravity, oxygen, etc) forces, and eukaryotes fending off prokaryotes, evolving the chemical balance of homeostasis and physiology. But you cannot defy the Laws of Physics forever. There has got to be ‘payback’ since matter and energy cannot be created or destroyed. Unicellular organisms are ‘immortalized’ by reproducing through binary fission, whereas eukaryotes ‘invented’ sexual reproduction as a means of communicating their genetic information from one generation to the next. If the ultimate evolutionary selection pressure for vertebrates is for reproductive success by reproduction, then the energy distribution throughout the ‘life cycle’ is not uniform, as in unicellular organisms, but instead it is biased in favor of the reproductive phase. The trade-off is that the cellular machinery must ultimately fail due to the omnipresence of bacteria, oxidative stress and other forces that initiated the evolutionary strategy. The result is decreased ‘energetics’ following the reproductive phase (Fig. 2), resulting in such phenomena as increased oxidative stress, lipid peroxidation, protein misfolding, endoplasmic reticulum stress, and failure of other such metabolic mechanisms in the process of aging. This then causes decreased cell communication as an energy requiring process, ultimately culminating in catastrophic failure of signaling, i.e. death. But the gene pool is ‘immortalized’ by the communication of DNA from one generation to the next. So, in the final analysis, each phase of this perspective on the ‘how and why’ of evolution is one of ‘cell communication’, initially between unicellular organisms and their physical environment, followed by cell communication as the basis for metazoan structure and function, and finally reproduction as the communication of genetic information from one generation to the next.

## Cell Communication is the Essence of Evolution

The cooperativity that underlies endosymbiosis in the emergence of eukaryotes has evolved from metabolic processes to cellular forms that have been recapitulated throughout the evolution of multicellular organisms as phylogeny and ontogeny. Take, for example, the epithelial-mesenchymal interactions that form tissues and organs. Such interactions are necessary for both the formation of the liver,^17^ as well as its homeostatic control of lipids, which shuttle back and forth between stellate cells and hepatocytes.^18^ The epithelial-mesenchymal cell-cell interactions that control development and regulation of endocrine tissues such as the adrenals, gonads, prostate and mammary gland can be viewed similarly.

In the cell communication model of lung development and homeostasis we have devised (Fig. 1), lipids maintain the structural integrity of the alveoli. Surfactant, a lipid-protein complex, is produced by epithelial type II cells in the corners of the alveoli. As lung volume increases and decreases with breathing, physical force (or stretch) on the alveoli regulates surfactant production and secretion. The connective tissue cells of the alveolar wall, or fibroblasts, actively recruit lipids from the circulation and transfer them to the epithelial type II cells for surfactant phospholipid synthesis.

Fibroblast lipid uptake and storage is mediated by adipocyte differentiation-related protein (ADRP), which is under the control of the Parathyroid Hormone-related Protein (PTHrP) signaling pathway. This series of functionally interrelated proteins is expressed compartmentally; PTHrP, surfactant, and the leptin receptor in the epithelium; PTHrP receptor, ADRP and leptin by the adjacent fibroblasts in the alveolar wall.

Interrupting this cellular homeostatic cross-talk causes epithelial and mesodermal cells to readapt in a process we recognize as disease.

It is hard to imagine that such a highly integrated and complex cell-molecular communication mechanism could have occurred purely by chance^19^-the appearance of an adipocyte-like cell-type in the alveolar wall, flanked by the vasculature on one side, the epithelium on the other, shuttling lipid from the circulation to the alveolar space under stretch-regulation by PTHrP and its receptor, leptin and its receptor, Prostaglandin E_2_ and its receptor. We speculate that for such a sequence of events to have occurred by chance would have taken more than the 5 billion years that the Earth has existed, if ever. Moreover, the fact that the direction and vectorial trajectory of lung ontogeny, phylogeny and pathophysiology (as reverse evolution)^11^ are all consistent with the evolution of this process (see Fig. 1) is hard to ignore. And because these fundamental relationships are linked by specific cell-molecular mechanisms, the model is experimentally testable and refutable.

Similar chains of events occur in all structures that ascribe to such developmental cell-cell interactions. The recognition that ontogeny, phylogeny, physiology and pathophysiology are a continuum of cell-cell communications infers that such motifs represent “rules” that could serve as guidelines for constructing a biologic periodic table.^20^

## Understanding Lung Evolution from the Middle-Out

The greatest challenge in the post-genomic era is to effectively integrate functionally relevant genomic data in order to derive physiologic first principles, and determine how to use them to decode complex physiologic traits. Currently, this problem is being addressed stochastically by analyzing large data sets to identify genes that are associated with structural and functional phenotypes-whether they are causal is largely ignored. This approach is merely an extrapolation of the Systema Naturae published by Linnaeus in 1735. The reductionist genetic approach cannot simply be computed to generate phenotype.^6,7,21^ Evolution is not a result of chance; it is an “emergent and contingent” process, just like the formation of the Universe. As Einstein famously stated, “God does not play dice with the Universe”.^22^ And ironically, Cosmologist Lee Smolin has applied Darwinian selection to stellar evolution, hypothesizing that there is a mechanistic continuum from elementary particles to the formation of Black Holes.

In our current and future research environment, we must expand our computational models to encompass a broad, evolutionary approach-as Dobzhansky^23^ has famously said, “Nothing in biology makes sense except in the light of evolution”. Elsewhere, we have formally proposed using a comparative, functional genomic, middle-out approach to solve for the evolution of physiologic traits.^24^ The approach engenders development, homeostasis and regeneration as a cluster of parallel lines that can be mathematically analyzed as a family of simultaneous equations.^8^ This perspective provides a feasible and refutable way of systematically integrating such information in its most robust functional genomic form to retrace its evolutionary origins (see Fig. 1). Among mammals, embryonic lung development is subdivided into two major phases: branching morphogenesis and alveolization. Fortuitously, we have observed that deleting the PTHrP gene results in failed alveolization.^25^ The generation of progressively smaller, more plentiful alveoli with thinning walls for gas exchange was necessitated by the selection pressure for the transition from water to land. This, and the fact that PTHrP and its receptor are highly conserved (the PTHrP ortholog Tuberoinfundibular Protein (TIP39) is expressed as far back in phylogeny as yeast), is stretch-regulated, and forms a paracrine signaling pathway mechanistically linking the endodermal and mesodermal germ layers of the embryo to the blood vessels, has compelled us to exploit this key transitional Gene Regulatory Network to further our understanding of physiology based on first principles.

This model transcends lung ontogenetic and phylogenetic principles. PTHrP produced by the lung epithelium regulates mesodermal leptin through a receptor-mediated mechanism. We have implicated leptin in the normal paracrine development of the lung, demonstrating its effect on lung development in the Xenopus tadpole,^26^ for the first time providing a functional, cell-molecular mechanism for the often-described and widely accepted co-evolution of metabolism, locomotion and respiration.^27,28^ These experiments have led to the question as to why the lipofibroblast appears in vertebrate lung alveoli starting with reptiles: the appearance of the lipofibroblast, an adipocyte-like mesenchymal derivative of the splanchnic mesoderm, could have evolved as an organizing principle for PTHrP/PTHrP receptor-mediated alveolar homeostasis as follows: muscle stem cells will spontaneously differentiate into adipocytes in 21% oxygen (room air) but not in 6% oxygen,^29^ suggesting that as the atmospheric oxygen increased over evolutionary time, lipofibroblasts may have formed spontaneously. Consistent with this hypothesis, we have previously shown that lipofibroblasts protect the lung against oxidant injury.^30^ Leptin is a ubiquitous product of adipocytes, which binds to its receptor in the alveolar epithelium of the lung, stimulating surfactant synthesis^31,32^ reducing surface tension, generating a progressively more compliant gas-exchange surface on which selection pressure could ultimately select for the stretch-regulated PTHrP co-regulation of surfactant and microvascular perfusion. This mechanism could have given rise to the mammalian lung alveolus, with maximal surface area resulting from stretch-regulated surfactant production and alveolar capillary perfusion,^33^ thinner alveolar walls due to PTHrP's apoptotic effect on fibroblasts,^34^ and a reinforced blood-gas barrier due to the evolution of type IV collagen.^35^ This last feature may have contributed generally to the molecular bauplan for the peripheral microvasculature of evolving vertebrates.

## The Cell Communication Model Guides us from current to Ancestral Phenotypes

We have more recently overarched the developmental and comparative aspects of the leptin mechanism by applying it to frog lung development. Crespi and Denver^36^ had shown that leptin stimulates tadpole limb development. This was an interesting observation because it provided a pleiotropic mechanism for the evolution of vertebrates, since metabolism, locomotion and respiration are the driving forces behind this process.^37^ To test the hypothesis that leptin biology might be a working model for vertebrate evolution, we treated Xenopus tadpole lung tissue with frog leptin, and, surprisingly, found that it had much the same effect that it does in mammalian lung-it stimulated thinning of the blood-gas barrier in combination with increased expression of surface-active phospholipid and proteins. The effects of leptin on surfactant were counterintuitive because the frog lung alveolus, termed a faveolus, is so large and muscularized that it does not require surface tension reducing activity physiologically. However, leptin has also been shown to stimulate Surfactant Protein A expression,^38^ which is an anti-microbial protein. This, and the fact that antimicrobial peptides are expressed in the gut^39^and skin^40^ suggests that the original selection pressure was for host defense, which was exapted for barrier expansion in the gut, lung and skin. That scenario is of interest in light of our studies of the effects of bacterial infection on lung development. We observed that the bacterial wall constituent lipopolysaccharide (lps) had the same stimulatory effect on developing lung epithelial cells that leptin has, suggesting that the original stimulus for the epithelial-mesenchymal interaction may have been due to extrinsic bacterial stimulation, followed evolutionarily by the intrinsic leptin mechanism. These interrelationships may relate back to the swim bladder origins of the lung, since the lung is homologous to the swim bladder of physostomous fish, which have a physical tube connecting the swim bladder to the gut, like a trachea, also creating access to the swim bladder for bacteria. This is in contrast to physoclistous fish, in which the swim bladder has no physical connection to the esophagus.

## Predictive Value of the Lung Cell Communication Model for Understanding the Evolution of Physiologic Systems

Unlike the classic pathophysiologic approach to disease, which reasons backwards from disease to health, the evolutionary-developmental approach, like Figure 2, reasons from the cellular origins of physiology, resulting in prediction of the cause of chronic disease, as we have shown for the lung^41^ and Fine has shown for the kidney.^42^ Fine specifically states that ‘Regeneration seems to follow the same pattern of sequential differentiation steps as nephrogenesis. The integrity of the epithelium is restored by reestablishing only those stages of differentiation that have been lost. Where cell death occurs, mitogenesis in adjacent cells restores the continuity of the epithelium and the entire sequence of differentiation events is initiated in the newly generated cells'.

As proof of principle, we will cite other examples of the fundamental difference between a pathophysiologic and an evolutionary approach to disease. As indicated above, we have found that PTHrP is a stretch-regulated gene that integrates the inflation and deflation of the alveolar wall with surfactant production and alveolar capillary perfusion. PTHrP is classically thought of as a bone-related gene that regulates calcium flux. With this and the stretch effect in mind, we recalled that astronauts develop osteoporosis due to weightlessness. Others have pursued a more conventional pathophysiologic tack to this phenomenon, reasoning that post-menopausal women develop osteoporosis and are estrogen deficient, therefore estrogen replacement would be the appropriate treatment for osteoporosis.^43^ We, on the other hand, have tested the hypothesis that microgravity would inhibit PTHrP expression in bone and lung cells. We observed a decrease in PTHrP expression by osteoblasts and lung epithelial cells in free-fall attached to dextran beads, mimicking 0 × g.^44^ When these cells were put back in unit gravity, the expression of PTHrP returned to normal levels in both cell-types. We subsequently examined the PTHrP expression by the weight-bearing bones of rats flown in deep space for 2 weeks.^44^ Here too, we observed a significant decrease in PTHrP expression compared to ground-based littermate controls. The weightlessness effect was not seen in the parietal bone, which is non-weight bearing, consistent with the effect of unloading of the weight bearing bones due to 0 × g.

We have also taken an unconventional evo-devo biologic approach to chronic lung disease. We have pursued the concept that there is an evolutionary continuum from development to homeostasis and regeneration mediated by soluble growth factors.^45^ Based on that approach, we have discovered that the cell communication between the epithelium and mesoderm is critically important for the development and maintenance of the alveolar lipofibroblast, and that when that signaling mechanism fails, the lipofibroblast ‘defaults’ to its cellular origin as a muscle cell, or myofibroblast, the signature cell-type for fibrosis. Not only has this approach given us insight to the multifactorial causes of Bronchopulmonary Dysplasia (pressure, oxygen, infection, maternal smoking (nicotine)), but to a novel treatment for BPD based on the use of Peroxisome Proliferator Activated Receptor gamma (PPARγ) as the nuclear transcription factor that determines the lipofibroblast phenotype. Thiazolidenediones are potent PPARγ agonists, and we have found that they can prevent^46^ or reverse the effects of all of the BPD-inducing agents we have studied,^47^ ranging from pressure to oxygen, infection and nicotine. This evolutionary-developmental approach may be far more successful in the treatment of BPD than more traditional, generic anti-inflammatory agents such as antenatal^48^ or postnatal steroids,^49^ or prophylactic surfactant therapy.^50^

As indicated earlier, evolutionary selection pressure generated metazoa through cell communication, leading to reproduction, aging and death, as shown in (Fig. 2). There is accumulating evidence for the loss of cell communication in aging rats^51–53^ to support this perspective on the life cycle. By inference, the selection pressure for reproductive success may optimize for cell communication. There is accumulating evidence in this regard as well.^54^ Therefore, one could devise strategies for ‘healthy aging’ based on this premise, rather than accepting the inevitability of aging as a slow, degradative pathologic process.^55^ It has recently been shown, for example, that there is a subset of aging humans who experience a precipitous death rather than experiencing the slow loss of biologic function over years.^56^ These data suggest that what we conventionally think of as aging and death is pathology, not evolved biology.

## Conclusion

With the help of the Human Genome, we must address the evolutionary origins of human physiology based on phylogenetic and developmental mechanisms. The approach we have proposed may fail to directly identify such first principles because we are missing intermediates from the ‘molecular fossil record’ that failed to optimize survival. But some aspects of those ‘failures’ were likely incorporated into other existing functional phenotypes, or into other molecularly-related functional homologies, like those of the lung and kidney, photoreceptors and circadian rhythms, the lens of the eye and liver enzymes. What this approach does provide is a robust means of formulating refutable hypotheses to determine the ultimate origins and first principles of physiology by providing candidate genes for phenotypes hypothesized to have mediated evolutionary changes in structure and/or function. It also forms the basis for predictive medicine^44^ and even for molecular bioethics, rather than merely showing associations between genes and pathology, which is unequivocally a Just So Story. In this new age of genomics, our reach must exceed our grasp.
